# Understanding the motivations, deterrents, and incentives for rural Albertan veterinary practice

**DOI:** 10.3389/fvets.2025.1633149

**Published:** 2025-09-18

**Authors:** Hanna Mughal, Thomas A. O’Neill, Lena Le Huray, Megan Bergman, John Remnant, Angelica M. Galezowski, Kent G. Hecker, Robert McCorkell

**Affiliations:** ^1^Department of Psychology, University of Calgary, Calgary, AB, Canada; ^2^Alberta Veterinary Medical Association, Edmonton, AB, Canada; ^3^Faculty of Veterinary Medicine, University of Calgary, Calgary, AB, Canada

**Keywords:** rural practice, rural attraction, rural retention, veterinary shortage, policy, education, practice

## Abstract

**Introduction:**

The shortage of rural veterinarians is a growing concern globally. This shortage increases the risk of significant negative impacts on livestock management, agriculture, and public health in rural and remote communities. To provide concrete solutions to sustain our rural veterinarian workforce, we examine motivations, incentives, and deterrents to rural veterinary practice (RVP). We do this through a qualitative study in Alberta, Canada, which is a geographically unique and understudied context.

**Methods:**

We surveyed veterinary students and practicing veterinarians, obtaining 124 responses. Data were analyzed using thematic analysis.

**Results:**

Results revealed key motivating factors that influence attraction and retention included personal and family considerations that require living in rural contexts, the nature of strong relationships that develop in rural communities, experiencing a range in work factors that enhances professional development, feeling fulfilled by rural veterinary work, and exposure during veterinary school leading to a strong interest in rural settings. Deterrents included limited resources and supports in rural contexts, personal and family needs that require living in urban settings, and challenges inherent to rural communities and environmental characteristics. Finally, key incentives included better salary and benefits, financial incentives, tuition/debt forgiveness, enhanced mentorship, fewer on-call duties, and tailored incentives.

**Discussion:**

Strong alignment between student and practicing veterinarian motivations, deterrents, and incentives was observed, extending previous findings that only look at the perceptions of a single group. The results corroborated previous findings, while revealing that the same motivations and deterrents remained important for students and PVs in Alberta’s geographically unique context. Finally, they provided key insights to inform policy, practice, and education developments to enhance attraction and retention rates of rural veterinarians, contributing to a path forward for addressing the rural shortage of veterinary services.

## 1 Introduction

There is a global shortage of veterinarians in rural contexts, as evidenced by high attrition rates of new graduates entering rural practice (1) and a decrease in rural interest over career progress (2). This trend is echoed in Canadian rural contexts and is reaching a crisis level (3). This reflects a systemic issue that rural Canadian communities consistently face regarding access to health care, childcare, talent attraction and retention, and affordable housing – all of which affect economic development in rural communities (4, 5). Veterinarians are a key component of this larger rural resource challenge.

The lack of rural veterinarians has significant implications for the health of animals and people within these communities and beyond. Practicing veterinarians (PVs) meet the needs of rural communities by providing services for both large and food animals, as well as the increasing number of companion pets (6, 7). Their impact reaches beyond rural communities; veterinarians play an integral role in food safety and public health and support the agricultural sector and therefore the economy (6, 7).

Moreover, the shortage may also negatively impact veterinarians who remain in rural practice; while community veterinary needs endure, remaining rural practitioners may need to increase their workload to accommodate this shortfall. Heavy workloads are a known source of stress and burnout in veterinarians (8) and can increase the likelihood of career attrition (9). In Alberta specifically, veterinarians are known to have the highest prevalence of veterinarians working long hours, suggesting heavy workloads (3) and an increased risk for burnout. The systemic impacts of the rural PV shortage demand attention as they put animal and public health at risk and further jeopardize the sustainability of rural veterinarian practice (RVP).

Despite this system-wide concern, only a handful of studies on the factors that influence attraction and retention rates for rural veterinarians have been conducted in Canadian contexts (2, 10, 11). Evidence from international contexts highlights motivating factors that influence the attraction of new graduates to RVP include: having a rural background (2, 7, 12); growing up working or living on a farm (2, 7, 12); previous experience and interest in large animals (7, 12); exposure to RVP during veterinary education (7, 13); and having a mentor from RVP (10, 14). Motivating factors that encourage experienced PVs to stay in RVP include strong ties with the community (13); strong relationships with clients (15, 16); supportive employers and a good work-life balance (15, 16); financial incentives (17); and opportunities for professional development (15).

Additionally, addressing deterrents and implementing incentives to rural practice may be another route to improving both attraction and retention rates. Potential deterrents to consider that have been highlighted in international studies include financial constraints (18, 19), high risk of injuries and burnout (9, 15), and work-life balance (15, 20). In turn, potential incentives from international studies include financial support (e.g., bonuses) (17), support for professional development (e.g., mentorship) (13, 15), and improved working conditions (e.g., support for work-life balance) (16, 17).

Despite these international findings, the need to uncover the current and most important motivating and deterring factors in Canadian rural veterinary contexts persists, and whether these factors differ meaningfully from findings in international settings that have different environments. We utilized the province of Alberta as a representative example of Canadian rural contexts and the challenges presented by Canadian geography, climate, and remote communities. Consulting the individuals at the center of this issue in Alberta is necessary to make recommendations to the Alberta Veterinary Association, governments, educators, and other partners regarding effective and impactful solutions. As such, our study aimed to achieve the following objectives: (1) understand the motivations for choosing a career in RVP; (2) identify deterrents preventing PVs and students from pursuing RVP; (3) and explore incentives to make RVP more attractive. The research question guiding this study was: *How can we attract and retain veterinary professionals in rural settings?* To achieve these objectives, we employed a mixed-method survey design, incorporating both demographic and open-ended questions to gather comprehensive insights.

## 2 Materials and methods

### 2.1 Sample

Participants consisted of both veterinary students and practicing veterinarians (PVs) in Alberta. Student participants were recruited from the University of Calgary Faculty of Veterinary Medicine and professional networks by an email sent by the Faculty to students, and PVs were recruited by an email sent by the Alberta Veterinary Medical Association (ABMVA).

### 2.2 Ethics statement

This research was approved by the University of Calgary Conjoint Faculties Research Ethics Board. Potential participants were provided prior informed consent, assured that their participation would be entirely voluntary, and that their data would be anonymous and confidential.

### 2.3 Measures

To assess the factors influencing attraction and retention in RVP, we relied on previous studies (21, 22) to develop two equivalent, parallel surveys that included both demographic questions and open-ended responses (see Supplementary Materials 1, 2). The demographic questions provided quantitative insights into the backgrounds, career paths, and current work environments of participants, whereas the open-ended questions allowed for a deeper exploration of their personal motivations, challenges, and experiences in RVP. The survey was piloted and tested internally with members of the research team.

### 2.4 Analysis

We utilized descriptive statistics to report on frequencies and demographics. In addition, we employed thematic analysis (23) to identify themes in the open-ended questions. The steps of thematic analysis include: (1) familiarizing with the data; (2) generating codes (building blocks of analysis); (3) constructing themes (clusters of codes that create meaning); (4) reviewing potential themes; (5) defining and naming themes, which was done through discussion by three of the authors; and (6) producing the report. We relied on an inductive approach with semantic and conservative coding. A primary coder first created the coding classifications, assigned codes, and discussed these with another researcher. Subsequently, a trained coder verified that codes were assigned accurately. Where discrepancies surfaced, they were resolved through discussion. Finally, we also employed a review process where a member of the research team reviewed half of the codes per theme and provided feedback. Consensus was reached on any points of disagreement.

## 3 Results

An invitation to complete the survey was sent to 159 University of Calgary Faculty of Veterinary Medicine students. There were 48 student responses (response rate = 30.19%). Furthermore, an invitation was sent to all members of the ABVMA by email listserv, containing approximately 2,390 practicing veterinarians (PVs). There were 76 practicing veterinarians who completed the survey (response rate = 3.18%). This generated 124 completed surveys that were included in the analysis.

### 3.1 Student demographics

Full demographic results for the 48 students can be found in Supplementary Material 3. Among the students surveyed, the majority were in their third year (46%), and almost all identified as white (79%) and as women (94%). Students’ practice intentions were divided into three roughly even groups: 40% intended to pursue rural practice, 33% intended to pursue urban practice, and 27% were undecided. Most students indicated they would like to focus on companion animals (40%) or mixed animal (33%) practice. Note that rural communities were defined as any community with a population equal to or below 24,999 residents.

### 3.2 PV demographics

Full demographic results for the 76 PVs can be found in Supplementary Material 3. Among the PVs surveyed, most had been practicing for over 10 years (75%), and most identified as white (96%) (Supplementary Table S1) and as women (70%). Most PVs worked in a rural setting (91%) and indicated they worked with mixed animals (48%).

**Figure 1:**
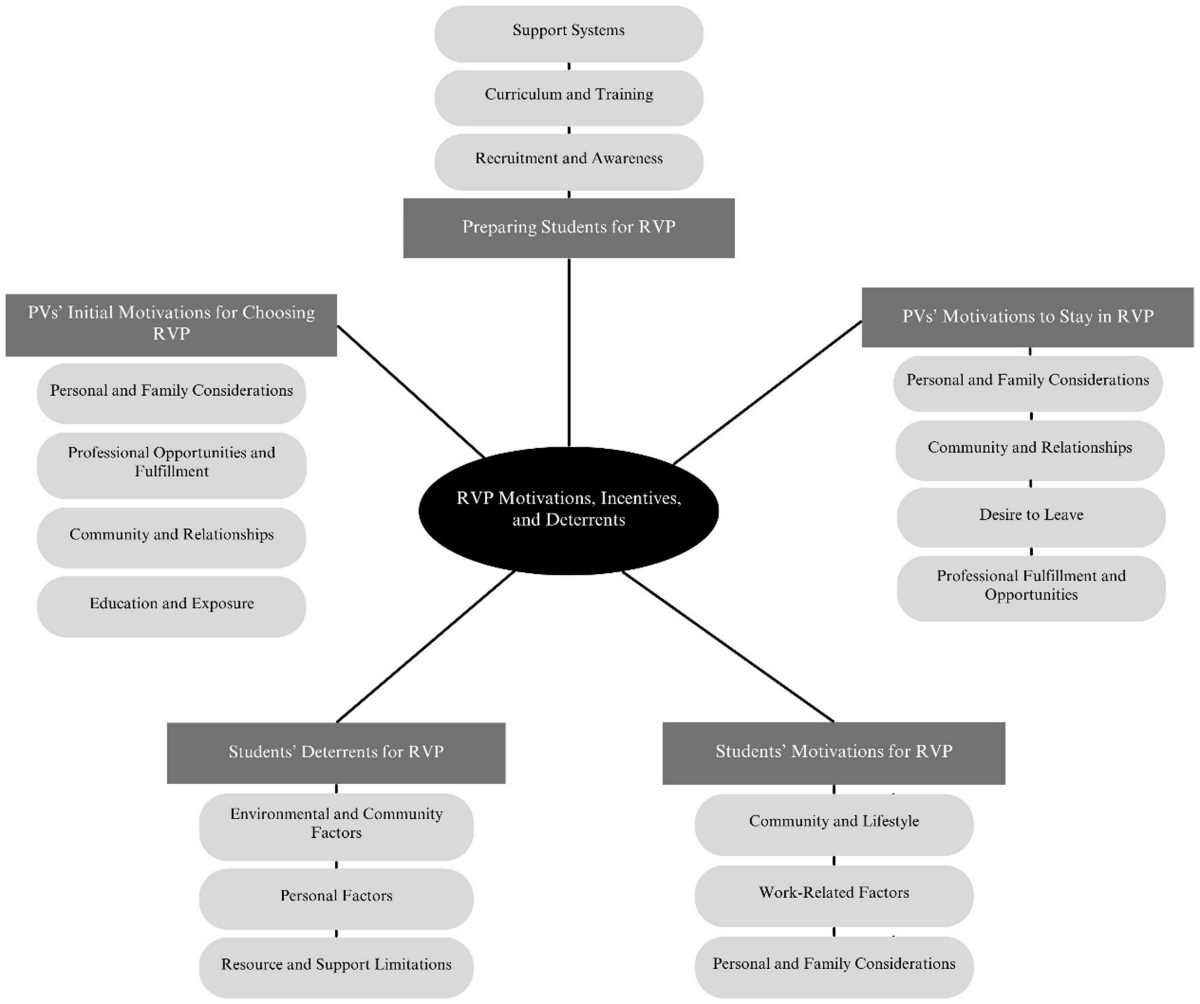
Topics and themes from qualitative data.

Below are the results of the thematic analysis by students and PVs (Figure 1).

### 3.3 Veterinary students’ results

#### 3.3.1 Motivations for RVP

The motivations to work in a rural setting of the students who intended to pursue rural practice generated three themes that included: (1) *community and lifestyle*, (2) *work-related factors*, and (3) *personal and family considerations*. The first theme, *community and lifestyle*, identified relationship-based and lifestyle factors that drew students to RVP, outlining that students desired to be part of a close-knit community where they felt a strong sense of belonging. Students felt a sense of duty to provide a needed service in these communities, seeing it as an opportunity to make a meaningful impact. They also noted how they appreciated the quieter, slower-paced lifestyle in rural settings, and financial freedom due to a lower cost of living.

*“I think the role we have as a veterinarian in these settings is very relationship-based. To me, this makes it a lot more rewarding. I also know that I one day want to raise my family in a rural community.”* (Theme 1).

The *work-related factors* theme highlighted professional aspects of RVP that were attractive to students. These included the preference to work with diverse animal populations typically found in rural practice, and the variety of cases and challenges were seen as valuable for professional and clinical skills development. In addition, the trusting and loyal relationships with clients that tend to develop in rural settings were attractive to students.

*“I wanted to work with a* var*iety of animals rather than just cats and dogs.”* (Theme 2).

Lastly, the *personal and family considerations* theme illustrated that prior rural experiences and having a rural background created a sense of familiarity with the lifestyle, prompting students to be more inclined to return to these contexts. A sense of belonging and family ties within rural communities aligned with this, also observed to be an important motivator.

*“I grew up in a rural community and I want to eventually [settle] down in that same community.”* (Theme 3).

#### 3.3.2 Deterrents for RVP

Twelve deterrents were identified for the remaining urban/undecided students who stated they had not considered a rural setting (Supplementary Material 3). The top five deterrents included: low rate of financial return for hours worked, on-call and after-hours demands, danger of working with large animals, location of job opportunities, and lack of social activities. To contextualize these results, three themes were generated, including: (1) *environmental and community factors*, (2) *personal factors*, and (3) *resource and support limitations.* The first theme, *environmental and community factors*, revealed that students’ perceptions of rural environments deterred them from rural practice. One important perception was the lack of diversity that could impact their personal and professional experiences. Students expressed concerns about encountering greater discrimination and different viewpoints compared to urban centers. Students also emphasized that entering rural practice would result in their removal from their current community and would significantly limit their access to social and recreation activities in urban centers.

*“Lack of diversity, fear of perceived bias or discrimination (especially as a neurodivergent female), different political ideologies, different views on animal use (especially in rodeos) …I think I’m too different to ever fit in or be accepted in the community.”* (Theme 1).

*Personal considerations*, including preferences for companion animals and personal values of animal treatment and welfare, presented in the second theme also influenced decreased interest in RVP.

*“I want to be a small animal surgeon and there would not be enough of a case load for me in a rural community.”* (Theme 2).

Lastly, *resource and support limitations* that are characteristic of rural settings acted as deterrents to RVP. Students expressed reservations about the lack of professional and mental health support in rural areas, and isolation. They were concerned about potential financial constraints due to lower income potential in rural areas compared to urban centers. Finally, the lack of infrastructure, services, and social and recreation activities was another factor that deterred students from RVP.

*“Lack of amenities and activities such as nice swimming pools, 24 h restaurants, nice restaurants, arts and culture, festivals, large and diverse population.”* (Theme 3).

#### 3.3.3 Incentives for students with RVP interest

A third (33%) of the students indicated they intended to pursue a career in an urban setting, while just under a third (27%) indicated they were undecided. Of the students who were undecided or intended to pursue an urban setting, 79% said they had considered rural practice, while 21% said they had not. Incentives are tangible offerings that can influence RVP considerations and intentions. As such, the top five incentives of the students who had not considered rural practice and who were undecided included: better salary and benefits, financial incentives, tuition/debt forgiveness, mentorship and additional support, and tailored incentives based on personal characteristics.

### 3.4 PV results

#### 3.4.1 Initial motivations for choosing RVP

Four themes were generated that represented the initial motivations to begin their career in rural practice of the PVs who currently work in a rural setting. These themes included: (1) *personal and family considerations*, (2) *professional opportunities and fulfillment*, (3) *community and relationships*, and (4) *education and exposure*. The first theme, *personal and family considerations*, revealed that personal considerations like preferences for a quiet lifestyle, quality of life inherent to rural contexts, and favoring the natural environment motivated PVs to choose RVP initially. Further, feelings of comfort with rural contexts due to growing up in rural areas or having positive past experiences were motivating factors. They expressed the desire to be close to family, to raise children in a rural environment, and to accommodate spousal employment.

*“Grew up in rural Alberta, all I knew about were mixed animal practices. It is my roots where I’m the happiest.”* (Theme 1).

Rural contexts offer unique *professional opportunities and fulfillment*, which were attractive to PVs. Such opportunities were described as diverse animals to treat, a broad scope of practice, a higher potential for ownership, higher job availability, more control over their work schedule, the ability to utilize advanced techniques, and cases that allowed PVs to develop a wide variety of clinical skills.

*“I wanted to practice large animal medicine and most of these practices are in more rural settings so following my career goals naturally lead toward a more rural environment.”* (Theme 2).

The third theme, *community and relationships*, highlighted that strong connections within the rural community was a strong motivator and was seen as essential for both professional success and personal satisfaction. The sense of belonging within the community, developed through the personal relationships with clients, co-workers, and community members, was another strong motivating factor for PVs. Further, the ability to make a meaningful impact by meeting the community’s veterinary needs in areas where veterinary services were limited or lacking was another important aspect of why PVs wanted to work in rural spaces initially.

*“I enjoy forming ties to the community and getting to know my clients (longer appointment times, farm visits, etc.).”* (Theme 3).

Lastly, the fourth theme, *education and exposure*, highlighted that gaining exposure through internships or rotations provided PVs with a realistic understanding of rural practice, reinforcing their interest and decisions to pursue a career in these settings. The rural interests and passions of professors and classmates also influenced PVs to want to practice rurally.

*“I began working in rural practices during my summers in veterinary school and developed an interest in the beef industry specifically.”* (Theme 4).

#### 3.4.2 Motivations to stay in RVP

In terms of the motivators that influenced rural PVs to remain in this setting, four themes were generated: (1) *personal and family considerations*, (2) *community and relationships*, (3) *professional fulfillment and opportunities*, and (4) *desire to leave*. *Personal and family considerations* in the first theme included a preference for the rural lifestyle and quality of life, love for the outdoors, and general enjoyment and satisfaction from living and working rurally. PVs valued the ability to raise children in rural areas, to stay close to family, and to be near partners’ jobs in the area.

*“…rural settings offer a freedom, privacy, and independence that is not easily fostered in urban settings.”* (Theme 1).

The second theme, *community and relationships*, underscored the importance of trusting and loyal relationships with clients, co-workers, and community members to maintain dedication to RVP. It was also seen as essential to professional success and personal satisfaction. Again, those who grew up rurally continued to feel a strong sense of connection and comfort in rural areas, further grounding them to the environment.

*“I’ve always loved working with livestock and poultry producers, as they work very hard to take care of their animals … They appreciate when we work with them to improve/resolve their disease outbreak situations they may be having.”* (Theme 2).

*Professional fulfillment and opportunities* highlighted in the third theme made it clear that the diversity in workload and animals found in rural practice, along with the chance to develop a range of skills, were important motivators. Increased opportunities for practice ownership, continuous skill development, and a steady flow of work providing job security were related motivators.

*“This type of practice is fantastic, rewarding (but also very challenging), and provides something different every day.”* (Theme 3).

Finally, the *desire to leave* did not go unacknowledged; some struggled to remain motivated, and challenges such as long work hours, on-call duties, and a desire to work more with small animals were referenced as factors that made PVs consider leaving.

*“Used to be passion for equine medicine, now finding it very hard to stay motivated to stay in equine practice, pay and hours and not sustainable long term to build a family and have quality of life…”* (Theme 4).

#### 3.4.3 Reasons for leaving

For the seven urban-based veterinarians who had left rural practice, there were several factors that influenced their decision to leave. One factor was the nature of referral practices for large animals, which are predominantly based in suburban areas. This geographic limitation made it challenging for veterinarians to find the necessary support and resources in rural settings. Additionally, personal and family considerations played a role. For example, one respondent highlighted the need to be closer to family as they planned to have children and required family support to manage both professional and personal responsibilities.

Another factor was related to employment conditions and support within the practice. Some veterinarians were asked to switch to part-time roles when new graduates were hired, likely due to overstaffing and the need to reduce hours. This was not financially viable for those who were the primary income earners, which forced them to seek new job opportunities elsewhere. Additionally, the lack of support from practice owners to reduce their workload and collaborative support from fellow veterinarians contributed to reported feelings of burnout and dissatisfaction. On-call duties, long work hours, and insufficient pay were commonly cited issues, likely creating challenges for managing family life and professional obligations. The absence of adequate mentorship and the strain of balancing on-call responsibilities with family life led many to seek positions in urban areas where support and resources were more readily available.

Incentives may have persuaded these PVs to stay. These top five incentives (from most to least important) were: fewer on-call duties, mentorship and additional support, financial incentives, better salary and benefits, and tailored incentives based on personal characteristics.

#### 3.4.4 Challenges for rural veterinarians

Challenges in rural contexts can impact rural PVs’ motivations. The top five challenges (from most to least) stated were: on-call and after-hours demands, physically tiring nature of working with large animals, dangers of working with large animals, low rate of return for hours worked, and issues with staff management (Supplementary Material 3). For those who chose “other,” the written responses included: lack of peer/colleague support, on-call pressures, referral challenges, hiring difficulties, client expectations and boundaries, poor salary, high workload, lack of anonymity, childcare, isolation, and technological limitations such as poor cell service in rural locations.

### 3.5 Preparing students for RVP

PVs were asked to provide recommendations on how veterinary schools can better prepare students for rural practice. From these data, three themes were generated: (1) *recruitment and awareness*, (2) *curriculum and training*, and (3) *support systems*. The first theme, *recruitment and awareness*, identified strategies to enhance admission and recruitment procedures to attract more individuals interested in RVP. Such strategies included targeting applicants with a rural background or passion for rural settings for admission and developing more realistic expectations of what rural practice entails throughout school, mainly in relation to the challenges and rewards such as on-call duties and the physically demanding nature of the work. Schools should also emphasize the unique benefits of RVP to students, including job opportunities, diversity of cases, and strong community relationships.

*“Focus on admitting students that are from a rural area. Interview students to see if they actually want to practice on large animals/in a rural area or are just saying what they need to on the application to make it seem like they do… Do not rely on just academic scores or MCAT…”* (Theme 1).

The second theme, *curriculum and training*, outlined a need for more comprehensive education and practical training to prepare veterinarians for future practice. Increased exposure to rural settings during school through internships, externships, and rotations, along with improving the knowledge and skills in students that are necessary to cover the full spectrum of care required in rural practice, were identified as necessary improvements to veterinary education. Strengthening the foundation of basic veterinary knowledge, in addition to offering advanced training opportunities, were additional suggested strategies. They believed that these approaches would improve interest and dedication to RVP.

*“I believe all students should have to do multiple rural placements prior to graduation. It allows them to see that there is more than just city life.”* (Theme 2).

Lastly, providing ongoing support to rural veterinarians was identified as a necessary method to bolster student preparation after graduation. Improving access to veterinary resources, increasing staff, and increasing wages were seen as strong steps forward to providing more assistance. Establishing mentorship programs to increase comfort and reduce isolation, plus fostering collaboration with rural colleagues, were all believed to be necessary supports after graduation to address retention rates.

*“When I graduated, mentorship was not a thing – you either swam at the front with everyone else, or you got out of the water…I think the awareness of the need to mentor new grads is much better now.”* (Theme 3).

## 4 Discussion

This study aimed to identify PVs’ and students’ perceptions of motivators and barriers to rural practice in Alberta, whether they aligned, and whether the unique geography of rural Alberta is related to different challenges when compared to other geographic locations. Most research on this topic focuses on either students *or* PVs. A novel contribution of this study was the investigation of both groups simultaneously, allowing us to compare their perceptions and experiences. Interestingly, there was consistent overlap between the motivations of students considering RVP, the initial motivations of PVs, and the motivations that support retention in RVP. Motivators identified by the participants reinforced previous findings (1, 11, 13, 15, 17, 19, 21, 22, 24). Notably, the importance placed on community and relationships by students and PVs suggests the importance of this factor (11). In addition, there was a moderate overlap in deterrents for both groups. Many deterrents identified by our study (e.g., on-call burdens, lack of resources) are corroborated by previous research (15, 16, 18, 19), emphasizing the continued need to address these concerns. Finally, both groups strongly agreed on incentives for rural practice, which suggests that the same incentives are likely effective in improving attraction and retention for both students and PVs, despite generational and other group differences. Overall, this strong alignment between the two groups is promising for interventions, as both groups are likely receptive to the same interventions.

These findings confirm that decisions of students and PVs in Alberta align with those of participants in published work in other contexts (13, 15–17), while also providing a rich descriptive summary of the ways these factors influence decision-making. Interestingly, the same motivators and deterrents appeared in our study as in previous research, despite Alberta’s unique setting. Rural Alberta is geographically very different than many of the settings where previous studies were conducted, which often included samples from countries in Western Europe. For instance, rural Alberta is often more remote, experiences more extreme weather patterns, and rural clients are more likely to require practitioners with a mixed practice skill set. Yet, despite these major contextual differences, students and PVs in Alberta highlight that they are motivated and deterred from rural practice for the same reasons as students and PVs in a Western European country. This finding reveals insight into the consistency of individual perceptions and experiences across varied geographies, resulting in important implications for the application of interventions across different locations.

### 4.1 Implications

There were several suggested strategies suggested by our results that may address some of the root issues with RVP attraction and retention. While untested in this study, these strategies – grounded in consultation with the people at the heart of the issue – provide opportunities for future pilots. Therefore, any strategies that are implemented must be rigorously evaluated to ensure that the intervention results in the desired outcomes.

First, the results suggest certain key educational opportunities that may help prepare students for RVP, including providing targeted hands-on experiences, coursework that covers the full spectrum of rural care required, and increasing exposure to rural environments through externships and rotations. Participants also believed that highlighting the unique benefits of rural practice may help attract students. While these strategies align with current efforts to improve the attraction and retention of rural health care practitioners (25), current evidence from the healthcare sector suggests that their effectiveness is mixed (26, 27). Thus, these strategies should be tested to identify their effectiveness.

Second, the findings highlighted the necessity for policy initiatives to address rural-specific challenges. Participants believed that policies that provide financial incentives, such as loan forgiveness programs, scholarships, and competitive salaries, may make rural practice more viable and attractive. Although aligned with similar initiatives in health care (4), evidence on the effectiveness of financial incentives in healthcare is inconsistent and warrants further investigation (26–29). Findings also suggest that mentorship programs and professional development opportunities may support retention rates, but testing is also required as there are limited studies to corroborate this approach (27).

Third, regarding practices, the results suggested several strategies that may improve retention rates, including fostering a supportive and inclusive work environment, reducing on-call demands, providing better access to resources and support systems, and promoting work-life balance. Building strong community connections and improving professional fulfillment were also seen as promising strategies to test. As noted, these strategies should be carefully evaluated as healthcare research does not provide any insight into their effectiveness (27). Finally, encouraging collaboration and teamwork within the practice may also help mitigate isolation and burnout. This approach is supported by research in healthcare and may be a rewarding solution to trial (29).

### 4.2 Limitations and future research directions

There is little evidence on the efficacy of potential strategies to address rural attraction and retention rates, even in the health care literature, so a crucial next step is to measure their actual impacts. Our study provides potential solutions to include in pilot applications that should be rigorously evaluated. Specifically, mentorship and relationships (in community and practices) emerged as strategies that should be prioritized in pilots.

Our findings also provided valuable insights from PVs who left rural for urban practice, however, the response rate from this group was low. Future research should further investigate their experiences to ensure that these insights are representative of all PVs who left for urban practice. Further, though our study was open to PVs in all contexts, we received no responses from PVs strictly in urban settings. Consulting this population in future research to understand their motivations and deterrents is crucial to address rural attraction.

Finally, gathering qualitative data through a survey may have presented limitations such as participants experiencing survey fatigue throughout the survey and/or from being frequently asked to complete surveys, in addition to surveys being a less engaging qualitative data collection method than interviews or focus groups. While we were able to gain detailed experiences and perceptions from a wide range of voices, we recognize that data from focus groups or interviews would strengthen and expand these findings. Future research should consider consulting students and PVs in focus groups and interviews.

## Data Availability

The de-identified aggregate data supporting the conclusions of this article will be made available by the authors, without undue reservation.
