# Supplementary material for: Understanding the motivations, deterrents, and incentives for rural Albertan veterinary practice
Source: Front Vet Sci. 2025 Sep 18;12:1633149. doi: 10.3389/fvets.2025.1633149 (PMC12488401; doi:10.3389/fvets.2025.1633149)
Supplement: Supplementary file 1 [file Supplementary_Materials.PDF]

# *Supplementary Material*

## 1 Supplementary Material 1

### Survey Questions for Veterinary Students

1. Are you a veterinary medicine student or a practicing veterinarian?
2. What year of the UCVM Doctor of Veterinary Medicine (DVM) program are you currently in?
3. Prior to your 18th birthday, where did you live? (select all that apply)
    a. On a farm
    b. A hobby farm
    c. An acreage
    d. A small rural community (pop. < 10,000)
    e. A medium rural community (pop. < 10,000 – 24,999)
    f. A small urban community (pop. < 25,000 – 49,999)
    g. A regional centre (pop. < 50,000 – 199,999)
    h. A metropolitan centre (pop. < 200,000)
4. Gender
5. Race
6. Age (in years) at the time you decided you wanted to be a veterinarian?
7. Current age (in years)?
8. Primary interest at entry into veterinary medical school?
9. Dependents?
10. What type of veterinarian practice setting are you intending to pursue?

If answer no to Q10:

11. Have you ever considered working in a rural setting?
12. What factors would make you consider a rural setting?
13. What are the main factors deterring you from considering a rural setting?
14. What are possible incentives that could persuade you to work in a rural setting?
15. Do you feel rural communities are welcoming to you? If no, please specify why.
16. What additional resources or training would you need ot feel prepared for rural practice?
17. What could the ABVMA do to better support the attraction and retention of rural veterinary professionals
18. Do you have anything else you would like to comment on in relation to…?
19. Would you be interested in participating in an online focus group?

End.

If answer yes to Q10:

11. What motivates you to consider working in a rural setting?
12. What type of animals do you want to devote most of your time to? (CA, FA, MA)
13. Do you feel your education has adequately prepared you to work in a rural setting?
14. Do you feel rural communities are welcoming to you? If no, please specify why.

15. What additional resources or training would you need ot feel prepared for rural practice?
16. What could the ABVMA do to better support the attraction and retention of rural veterinary professionals
17. Do you have anything else you would like to comment on in relation to…?
18. Would you be interested in participating in an online focus group?

End.

## 2 Supplementary Material 2

### Survey Questions for Practicing Veterinarians

1. Are you a veterinary medicine student or a practicing veterinarian?
2. How many years have you been in practice?
3. What is your level of practice ownership?
4. Prior to your 18th birthday, where did you live? (select all that apply)
    a. On a farm
    b. A hobby farm
    c. An acreage
    d. A small rural community (pop. < 10,000)
    e. A medium rural community (pop. < 10,000 – 24,999)
    f. A small urban community (pop. < 25,000 – 49,999)
    g. A regional centre (pop. < 50,000 – 199,999)
    h. A metropolitan centre (pop. < 200,000)
5. Gender?
6. Race?
7. Age (in years) at the time you decided you wanted to be a veterinarian?
8. Current age (in years)?
9. Primary interest at entry into veterinary medical school?
10. Dependents?
11. What was your initial career environment?
12. What is your current career environment?

If answered urban to Q12:

13. Have you ever worked in a rural setting?

If answered yes to Q13:

14. What factors caused you to leave?
15. What incentives could have persuaded you to stay working in a rural setting?

Return to last set of questions for all.

If answered no to Q13:

16. What are the main factors that deterred you from working in a rural setting?
17. What are the possible incentives that could have persuaded to you work in a rural setting?

Return to the last set of questions for all.

If answered rural to Q12:

18. What motivated you to choose RVMP initially?
19. What type of animals do you devote most of your time to? (CA, FA, MA)
20. What are the main factors that motivate you to stay working in a rural setting?
21. What are the main challenges that you face as a rural veterinarian?

Return to the last set of questions for all.

Last set of questions for all:

22. Do you feel integrated within your rural community? Please elaborate.
23. What recommendations would you make to veterinarian schools to better prepare students for rural practice?
24. What can the ABVMA do to better support rural veterinary retention and recruitment?
25. Do you have anything else you would like to comment on in relation to attracting, working, and retaining…?
26. Do you wish to be entered into a draw for a prize?
27. Would you be interested in participating in an online focus group?
28. Would you like to be contacted for a focus group?

End.

## 3 Supplementary Material 3: Supplementary Tables

### 3.1 Table 1

*Student Sample Demographics*[a]

| Demographic | *n* | % |
|---|---|---|
| Year of study | 10 | 21 |
| Second year | 22 | 46 |
| Third year | 16 | 33 |
| Fourth year | | |
| Racial/ethnic identification | | |
| White | 38 | 79 |
| First Nations (Indigenous, Métis, Inuit) | 3 | 6 |
| Southeast Asian | 2 | 4 |
| South Asian | 2 | 4 |
| Southeast Asian and white | 1 | 2 |
| Middle Eastern | 1 | 2 |

| | | |
|---|---|---|
| East Asian | 1 | 2 |
| Gender | | |
| Woman | 45 | 94 |
| Man | 3 | 6 |
| Environment during upbringing | | |
| Farm | 20 | 22 |
| Hobby farm | 6 | 7 |
| Acreage | 9 | 10 |
| Small rural community | 19 | 21 |
| Medium rural community | 6 | 7 |
| Small urban community | 4 | 4 |
| Regional centre | 10 | 11 |
| Metropolitan centre | 17 | 19 |
| Primary practice interest of students | | |
| Companion animal-exclusive practice | 19 | 40 |

| | | |
|---|---|---|
| Mixed-animal practice | 16 | 33 |
| Equine practice | 5 | 11 |
| Food animal-exclusive practice | 4 | 8 |
| Zoo, wildlife, or exotic animal practice | 2 | 4 |
| Other | 2 | 4 |

[a] N = 48.

### 3.2 Table 2

*PV Sample Demographics*[a]

| Demographic | *n* | % |
|---|---|---|
| Racial/ethnic identification | | |
| White | 73 | 96 |
| First Nations (Indigenous, Métis, Inuit) | 1 | 1 |
| Another race category | 1 | 1 |
| Prefer not to say | 1 | 1 |
| Gender | | |

| | | |
|---|---|---|
| Woman | 53 | 70 |
| Man | 23 | 30 |
| Environment during upbringing | | |
| Farm | 41 | 30 |
| Hobby farm | 8 | 6 |
| Acreage | 11 | 8 |
| Small rural community | 42 | 31 |
| Medium rural community | 7 | 5 |
| Small urban community | 5 | 4 |
| Regional centre | 7 | 5 |
| Metropolitan centre | 16 | 12 |
| Years of practice | | |
| 1-5 years | 10 | 13 |
| 6-10 years | 9 | 12 |
| More than 10 years | 57 | 75 |

| | | |
|---|---|---|
| Current practice setting | 4 | 8 |
| Rural | 69 | 91 |
| Urban | 7 | 9 |
| Level of practice ownership | | |
| Employee | 40 | 52 |
| Partner | 15 | 20 |
| Sole owner | 21 | 28 |
| Animals primarily work with | | |
| Companion/small-animal only | 11 | 16 |
| Food-animal only | 16 | 23 |
| Mixed-animal | 33 | 48 |
| Equine | 5 | 7 |
| Other | 4 | 6 |

[a] N = 76.

### 3.3 Table 3

*Comparison of Factors that Deter Students from RVMP with Factors Reported as Challenges by Rural PVs*

| Factors | Students | PVs |
|---|---|---|
| On-call and after-hours demands | 13.61% | 18.06% |
| Low rate of return for hours worked | 14.28% | 9.67% |
| Dangers of working with large animals | 11.56% | 10% |
| Lack of social activities | 10.20% | 4.33% |
| Other | 8.16% | 6.45% |
| Employment opportunities for spouse and family members | 9.52% | 4.33% |
| Inadequate medical services | 6.12% | 5.00% |
| Reliable internet services | 3.40% | 1.33% |
| Cost of living | 3.40% | 1.00% |
| Lack of sporting communities | 2.04% | 1.00% |
| Location of job opportunities | 10.88% | NA |
| Physically demanding nature of work | 6.80% | NA |
| Physically tiring nature of working with large animals | NA | 10.67% |
| Issues with staff management | NA | 10.67% |
| Difficulties with clients | NA | 4.67% |
| Lack of mentorship and support | NA | 4.33% |
| Lack of equipment | NA | 1.33% |

| | | |
|---|---|---|
| Major disease outbreaks | NA | 0.33% |

*Note*. NA = response option not available on survey. Percentages calculated separately for students and PVs by dividing the number of votes for one response by the total votes across all response options (participants could select multiple responses).
